# Increased Parasympathetic Activity by Foot Reflexology Massage after Repeated Sprint Test in Collegiate Football Players: A Randomised Controlled Trial

**DOI:** 10.3390/sports7110228

**Published:** 2019-11-03

**Authors:** Yung-Sheng Chen, Wan-An Lu, Filipe Manuel Clemente, José Pedro Bezerra, Cheng-Deng Kuo

**Affiliations:** 1 Department of Exercise and Health Sciences, University of Taipei, Taipei 111, Taiwan; yschen@utaipei.edu.tw; 2 Institute of Cultural Asset and Reinvention, Fo-Guang University, Yilan 262, Taiwan; wanan.lu@msa.hinet.net; 3 Escola Superior de Desporto e Lazer, Instituto Politécnico de Viana do Castelo, 4960-320 Melgaço, Portugal; filipe.clemente5@gmail.com (F.M.C.); pedro.bezerra62@gmail.com (J.P.B.); 4 Instituto de Telecomunicações, Delegação da Covilhã, 1049-001 Lisboa, Portugal; 5 The Research Centre in Sports Sciences, Health Sciences and Human Development, 5001-801 Vila Real, Portugal; 6 Division of Chest Medicine, Department of Internal Medicine, Changhua Christian Hospital, Changhua 500, Taiwan; 7 Department of Medical Research, Taipei Veterans General Hospital, Taipei 112, Taiwan

**Keywords:** foot reflexology massage, parasympathetic activity, intermittent exercise, vagal tone

## Abstract

Foot reflexology massage (FRM) has positive effects on cardiovascular and haemodynamic functions. However, information regarding the physiological changes after FRM post exercise-stress is limited. This study investigated the acute effects of FRM on heart rate variability (HRV) after the repeated sprint ability (RSA) test and the Yo-Yo Intermittent Recovery Test Level 1 (YY). Twenty-six collegiate male football players were randomly assigned to the FRM group (n = 14) or to the control group (n = 12). Electrocardiographic (ECG) signals were recorded for 15 min in supine position before and after the intervention/control period in the RSA test and the YY test. In comparison to the control group, the FRM group demonstrated higher values of root mean squared successive difference in the RR interval (RMSSD; *p* = 0.046, ES = 0.76) and in the proportion of differences of adjacent RR intervals >50 ms (pNN50; *p* = 0.031, ES = 0.87); and higher percent changes in mean RR interval (%MeanRR; *p* = 0.040, ES = 0.99), standard deviation of RR intervals (%SDNN; *p* = 0.008, ES = 1.10), normalised high-frequency power (%nHFP; *p* = 0.008, ES = 0.77), total power (%TP; *p* = 0.009, ES = 0.84) and standard deviation 1 and 2 (%SD1; *p* = 0.008, ES = 1.08, %SD2; *p* = 0.020, ES = 1.04) after the RSA test. The magnitude effect of post-exercise HRV was small after the FRM RSA protocol (ES = 0.32–0.57). Conversely, the results demonstrated a moderate and large magnitude effect of HRV in the RSA and YY protocols of the control group (ES: RSA = 1.07–2.00; YY = 0.81–1.61) and in the YY protocol of the FRM group (ES = 0.99–1.59). The FRM intervention resulted in beneficial effects on the cardiac parasympathetic reactivity and the sympatho-vagal balance after RSA performance.

## 1. Introduction

Recovery strategies are a primary concern in sports sciences and medicine. Massage is a popular recovery modality after training sessions and competitions [1]. Post-exercise sports massage can alter molecular responses in relation to inflammation (i.e., leucocyte demargination) [1], creatine kinase levels, improvement of maximal isometric strength [2], muscle oedema, pain, and muscle spasm [3,4]. In contrast, the impairment of the massage effect on blood flow and lactate acid removal was observed after a 2-min continuous handgrip isometric contraction exercise [5]. Non-significant changes in the massage effect on blood lactate (BL) removal and punching performance were found in boxers after 5 sets of 2-min repeated bouts of boxing punching [6]. The controversial evidence of massage intervention may be related to differences in massage technique, duration of intervention, body area being massaged and type of testing across studies [7].

Reflexology treatment requires a specific massage technique applied to the reflex areas mainly in the upper and lower extremities [8]. Practitioners stimulate the reflex points, resulting in physiological feedback via a reflex loop to ‘mapped target organs’. The theoretical background proposed by reflexologists is based on haemodynamic and nerve impulse properties [9]. The acute effects of reflexology massage on blood pressure, heart rate (HR) patterns, and salivary cortisol concentration have been reported [10,11]. Lu, Chen and Kuo [12] demonstrated that vagal activation was increased while sympathetic modulation and arterial blood pressure were decreased after a 60-min foot reflexology massage (FRM). The physiological changes in autonomic nervous function are due to the enhancement of vagal tone modulation via stimulating a neural baroreflex during the FRM intervention. Parasympathetic reactivation is considered as an essential factor for modulating cardiovascular function during exercise recovery because of homeostasis [13].

Football requires anaerobic- and aerobic-based capacities to achieve the athletic demands of the matches [14]. Chen et al. [15] reported that intermittent anaerobic and aerobic exercises contribute to different physiological strains on the autonomic nervous system in football players. However, whether these physiological strains can be alleviated by a FRM intervention is unclear. The aim of this study is to examine the acute effects of FRM on the modulation of heart rate variability (HRV) after anaerobic and aerobic-based strenuous exercises, repeated sprint ability (RSA) and Yo-Yo Intermittent Recovery Test Level 1 (YY) tests, respectively, in collegiate football players. The rationale to adopt FRM rather than hand reflexology massage is that football players mainly use muscle activation of the lower extremities (i.e., running, jump, kicking a ball, etc.) during training sessions and competitions. We hypothesised that a significant difference in HRV after RSA and YY would be observed in the FRM group, but not in the control group.

## 2. Materials and Methods

### 2.1. Participants

**Figure 1:**
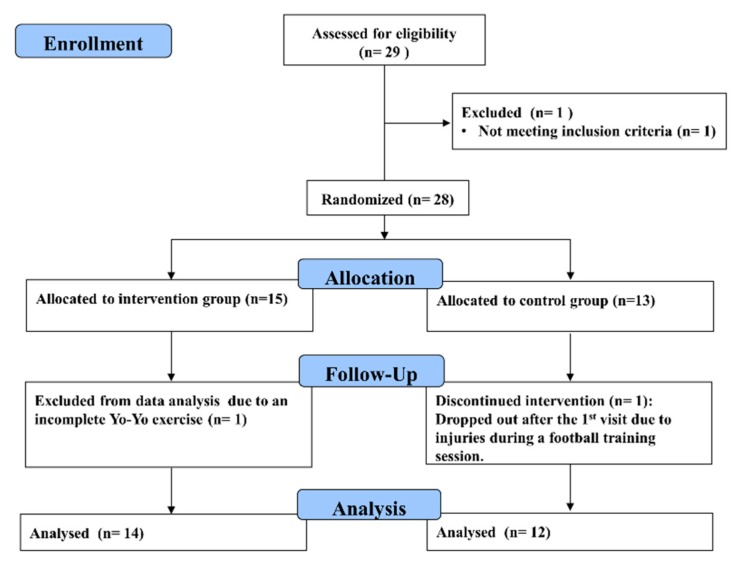
CONSORT flow diagram of the study. One participant was excluded from data analysis because of uncompleted Yo-Yo test.

Twenty-six collegiate male football players were recruited in this study. The participants underwent football training at a frequency of 3–5 times per week, and the weekly training time was within a range of 8–12 h (2–2.5 h per training session). All participants were assigned to either the intervention group (n = 14; age: 19.3 ± 1.0 years, height: 172.9 ± 4.5 cm, weight: 68.7 ± 6.2 kg, years of playing experience: 10.1 ± 1.8 years) or the control group (n = 12; age: 20.9 ± 1.0 years, height: 173.3 ± 4.3 cm, weight: 65.3 ± 5.4 kg, years of playing experience: 10.1 ± 3.2 years) using a computer-generated random number table (https://www.randomizer.org/) after confirmation that there are no differences between the groups in the variables (Figure 1). To avoid the sham effect of reflexology treatment, the participants in the intervention group were required to have no experience with FRM. The exclusion criteria included history of severe neuromuscular injury, current lower extremity injury and neurological diseases. The participants signed a written informed consent form and underwent a familiarisation session before the experiment. This study was approved by the human ethics committee of the University of Taipei (UT-IRB-2016-017), registered at the ClinicalTrials.gov with the identifier NCT03821805, and was conducted in accordance with the Declaration of Helsinki.

### 2.2. Sample Size Estimation

The sample size estimation was determined using G*Power 3.1.9.4 software (G*Power, Düsseldorf, Germany) [16]. Based on our previous study [15], a power of 80% and an alpha value of 0.05 in the two-tailed test were set to estimate the minimum number of participants. The power analysis indicated that a minimum of 14 participants in the intervention group would be required.

### 2.3. Experimental Procedure

The participants were requested to avoid strenuous exercise 24 h before the experiment and to refrain from caffeine-containing substances and smoking 2 h before the experiment. The participants were required to lie supine in a quiet research room to obtain electrocardiographic (ECG) records (MP35, Biopac Inc., Goleta, CA, USA) via conventional lead II arrangement. A portable Polar HR monitor (RS800CX; Polar Electro, Kemple, Finland) was mounted onto the participants’ front chest to record the HR during exercises. Baseline ECG signals were recorded in a supine position for 15 min, followed by pre-exercise BL concentration measurement (h/p/Cosmos Sirius; SensLab, Leipzig, Germany). The first 5-min ECG data were discarded to prevent orthostatic effect. Subsequently, the participants performed a 5-min 50-watt cycling exercise for warm-up activity. The second BL sample was taken immediately after the YY and RSA exercises. The participants in the FRM group were given a 30-min FRM treatment after the exercises. In contrast, the participants in the control group were allowed to rest on a massage table for 30 min. Post-exercise ECG signals were then recorded in supine position for 15 min. The participants performed the YY and RSA exercise protocols indoors on artificial lanes in two different occasions with at least two days apart. All experimental sessions were conducted between 08:00 and 13:00. Room temperature was controlled at 25 °C, and humidity was set within the range of 50 to 60%.

### 2.4. Reflexology Intervention

The foot reflexology technique developed by Father Josef was used as the massage intervention in this study. The FRM was performed first on the non-dominant foot and then on the dominant foot while the participants were lying on a massage table. The FRM was directed to the reflex zones and included the area of the toes, sole, heel, ankle and calf muscles. Massage oil was applied to the skin during the FRM. The FRM technique processes consisted of three parts: (1) Preparation phase (2 min): skin clean, hot pack, rub and knead; (2) operational Phase (12 min): clamping by index finger and thumb finger, push by finger pulp and slip by index finger joint (basis phalanges); and (3) integrative phase (1 m in): buckle, traction, clean. The FRM was performed 15 min for each foot by a qualified foot reflexologist with 20 years of experience from the Taiwan Association of Reflexology.

### 2.5. Exercise Protocols

#### 2.5.1. YY Intermittent Recovery Test Level 1

The YY test consisted of 10 s active recovery after each bout of interval running, jogging distance of 5-min recovery zone and repeated 20-min runs back and forth between the start and return lines, with gradually incremental speed [17]. The speed of interval running was controlled by digital audio bleeps from a laptop. The speed of interval running was 10–13 km·h^−1^ (0–160 m) in the first four bouts and was 13.5–14.0 km·h^−1^ (160–440 m) in another seven bouts. The speed was then increased by 0.5 km·h^−1^ after every eight bouts (i.e., after 760, 1080, 1400, 1720 m, etc.). The total covered distance was recorded when the participants failed to reach the start line in time twice.

#### 2.5.2. Repeated Sprint Ability Test

The RSA test consisted of 20-m interval sprint repeated 6 times with 20-s rest interval [18]. Two preliminary trials were allowed to all participants to familiarise with the RSA, followed by a 5-min rest. A time gate system (Fusion Sport, Coopers Plains, Australia) was aligned at the starting line to record the sprint time.

### 2.6. Measurements

#### 2.6.1. Heart Rate Variability

The analog signals of ECG were transformed into digital signals using an analog-to-digital converter with a sampling rate of 1000 Hz. The ECG waveforms were then filtered using a commercial HRV analysis software (Premium version 3.0, Kubios, Kuopio, Finland) to calculate the HRV indices. The R-R intervals (RRI) were calculated after eliminating the ectopic beats. If the percentage of ectopic beats was greater than 5%, data from the participant were excluded from the analysis.

The mean RR (MeanRR), standard deviation of RR (SDNN), mean sum of the squared differences between RR (RMSSD) and proportion of NN50 count divided by the total number of all RRs (pNN50) were calculated by using the standard formulae for time domain analysis. The power spectra of RR were calculated by means of fast Fourier transformation for frequency domain analysis. The areas under the spectral peaks within the ranges of 0.01–0.4 Hz, 0.04–0.15 Hz and 0.15–0.4 Hz were defined as the total power (TP), low-frequency power (LFP) and high-frequency power (HFP), respectively [19]. The normalised power of LFP and HFP (nLFP and nHFP) were used to calculate the powers of frequency bands in normalised units [20]. The low-/high-frequency power ratio (LFP/HFP) was used as the index of sympatho-vagal balance. Nonlinear analyses of the Poincaré plot indices SD1 and SD2 were also performed to determine the nonlinear characteristics of HRV.

#### 2.6.2. Blood Lactate Measurement

Blood samples were taken from the middle fingertip before and immediately after the completion of the exercise. The area of blood withdrawal was cleaned with an alcohol swab, and a blood sample was drawn into the test strip.

#### 2.6.3. Rating of Perceived Execution

A conventional 15-point Borg scale was used to evaluate the rate of perceived exertion (RPE) [21]. The participants reported the rate of RPE immediately after completing the first lap and last lap of the exercises.

### 2.7. Data Analyses

The total, best, worst and decrement rate of RSA performance were recorded as RSA_total_, RSA_fastest_, RSA_slowest_ and RSA_decrement_, respectively. The RSA_total_ was calculated as the sum of six sprint times. The RSA_fastest_ showed the best performance in the six sprints, whereas the RSA_slowest_ showed the worst performance in 6 sprints. The RSA_decrement_ was calculated by using the following formula: RSA_decrement_ (%) = {1 − [RSA_total_/(RSA_fastest_ × 6)]} × 100 [22].

To avoid daily variation of physical status, the pre-and-post exercise percent change in HRV measures after the exercise were calculated by using the following formula [15]:

%X = [(X_after_ − X_before_)/X_before_] × 100%

where “X” denotes the HRV measures to be evaluated.

### 2.8. Statistical Analyses

Descriptive data were presented as median values with interquartile range. Statistical analyses were conducted using Sigmaplot version 13 for Windows (Sigmaplot, Systat Software, San Jose, CA, USA). The Mann-Whitney rank sum test was used to evaluate the differences in the between-group comparison in physical characteristics, exercise performance, BL, RPE and HRV indices. The Wilcoxon signed rank test was used to evaluate the within-group comparison (time frames) in BL, RPE and HRV indices. An effect size (ES) was calculated to evaluate the magnitude of an intervention effect. The magnitude of ES was defined as follows: trivial (ES < 0.2), small effect (ES = 0.2–0.6), moderate effect (ES = 0.6–1.2), large effect (ES = 1.2–2) and very large effect (ES > 2.00), according to guidelines suggested by Hopkin et al. [23]. The alpha level of significant difference was set at *p* < 0.05.

## 3. Results

### 3.1. Exercise Performance

Group comparison showed no significant differences in RSA_total_ (*p* = 0.449, ES = −0.23), RSA_slowest_ (*p* = 0.317, ES = −0.42), RSA_fastest_ (*p* = 0.542, ES = −0.14), RSA_decrement_ (*p* = 0.826, ES = 0.32) and YY total covered distance (*p* = 0.074, ES = 0.61). Exercise performance RSA and YY exercise protocols are presented in Table 1.

### 3.2. Rating of Perceived Exertion, Blood Lactate and Exercise Peak Heart Rate

The pre- and post-exercise BL concentrations, peak HR, first and last laps of RPE during exercises were not statistically different between groups during the RSA and YY exercise protocols (*p* > 0.05; Table 1).

### 3.3. Heart Rate Variability

**Figure 2:**
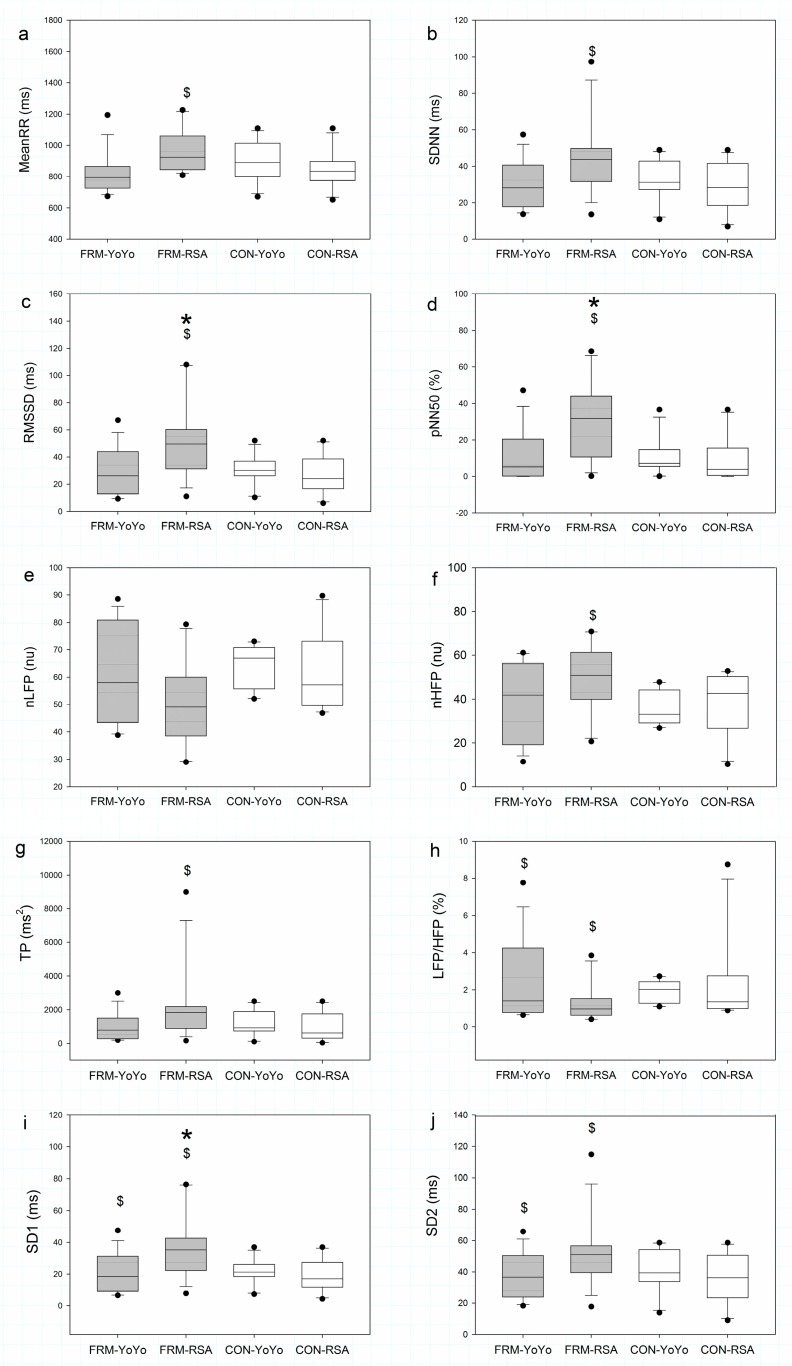
Post-exercise measure of heart rate variability modulation in the foot reflexology massage (grey colour) and control (white colour) groups during Yo-Yo Intermittent Recovery Test Level 1 and repeated sprint ability test. MeanRR = Mean RR interval; SDNN = standard deviation of RR interval; RMSSD = the mean sum of the squared differences between RR intervals; pNN50 = NN50 count divided by the total number of all RR intervals; nLFP = normalised low-frequency power; nHFP = normalised high-frequency power; TP = total power; SD1 = the standard deviation of the points perpendicular to the line of symmetry; SD2 = the standard deviation of the points along the line of symmetry. * Significant difference between groups (*p* < 0.05). $ Significant difference between exercise protocols (*p* < 0.05). (**a**) MeanRR; (**b**) SDNN; (**c**) RMSSD; (**d**) pNN50; (**e**) nLFP; (**f**) nHFP; (**g**) TP; (**h**) LFP/HFP ratio; (**i**) SD1; (**j**) SD2.

The baseline HRV indices were not significantly different in both groups. However, the result of post-exercise comparison showed significant differences between exercise protocols in the FRM group (Figure 2). The MeanRR (*p* = 0.007, ES = 0.86), SDNN (*p* = 0.030, ES = 0.73), RMSSD (*p* = 0.025, ES = 0.78), pNN50 (*p* = 0.020, ES = 0.84), TP (*p* = 0.042, ES = 0.66), LFP/HFP (*p* = 0.025, ES = −0.35), SD1 (*p* = 0.025, ES = 0.78) and SD2 (*p* = 0.049, ES = 0.67) in the FRM group were significantly higher after the RSA exercise than after the YY exercise. By contrast, no significant difference was found in HRV indices between the exercise protocols and control groups (*p* > 0.05). Group comparison in the post-exercise measurement showed that the FRM group had higher values for RMSSD (*p* = 0.046, ES = 0.76), pNN50 (*p* = 0.031, ES = 0.87), and SD1 (*p* = 0.046, ES = 0.76) than the control group after RSA exercise.

**Figure 3:**
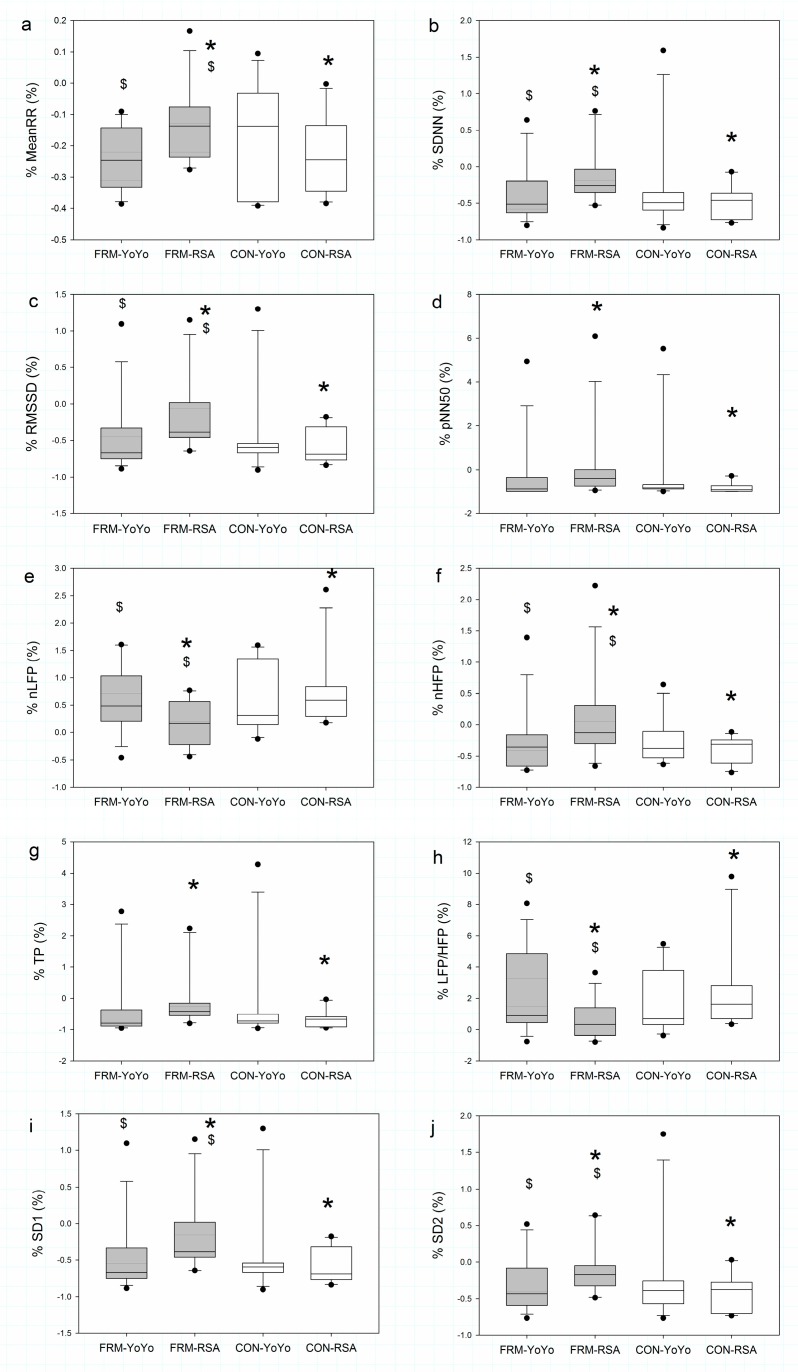
Comparison of the pre-and-post- exercise percent change in heart rate variability indices after the Yo-Yo Intermittent Recovery Test Level 1 and Repeated Sprint Ability test in the foot massage treatment (grey colour) and control (white colour) groups. MeanRR = mean RR interval; SDNN = standard deviation of RR interval; RMSSD = the mean sum of the squared differences between RR intervals; pNN50 = NN50 count divided by the total number of all RR intervals; nLFP = normalised low-frequency power; nHFP = normalised high-frequency power; TP = total power; SD1 = the standard deviation of the points perpendicular to the line of symmetry; SD2 = the standard deviation of the points along the line of symmetry. * Significant difference between groups (*p* < 0.05). $ Significant difference between exercise protocols (*p* < 0.05). (**a**) %MeanRR; (**b**) %SDNN; (**c**) %RMSSD; (**d**) %pNN50; (**e**) %nLFP; (**f**) %nHFP; (**g**) %TP; (**h**) %LFP/HFP ratio; (**i**) %SD1; (**j**) %SD2.

In the comparison of pre-and-post exercise percent change, the FRM group demonstrated higher %MeanRR (*p* = 0.040, ES = 0.99), %SDNN (*p* = 0.008, ES = 1.10), %pNN50 (*p* = 0.008, ES = 0.66), %RMSSD (*p* = 0.008, ES = 1.08), %nHFP (*p* = 0.008, ES = 0.77), %TP (*p* = 0.009, ES = 0.84), %SD1 (*p* = 0.008, ES = 1.08) and %SD2 (*p* = 0.020, ES = 1.04) and were lower in %nLFP (*p* = 0.013, ES = −0.93) and %LFP/HFP (*p* = 0.009, ES = −0.79) than those in the control group during the RSA exercise. In the exercise comparison, the FRM group demonstrated higher values for %MeanRR (*p* = 0.004, ES = 1.09), %SDNN (*p* = 0.017, ES = 0.73), %RMSSD (*p* = 0.002, ES = 0.69), %nHFP (*p* = 0.030, ES = 0.54), %SD1 (*p* = 0.002, ES = 0.69) and %SD2 (*p* = 0.035, ES = 0.70), and lower values for %nLFP (*p* = 0.020, ES = −0.87) and %LHR (*p* = 0.007, ES = −0.92) during the RSA exercise protocol, compared to the YY exercise protocol (Figure 3).

**Figure 4:**
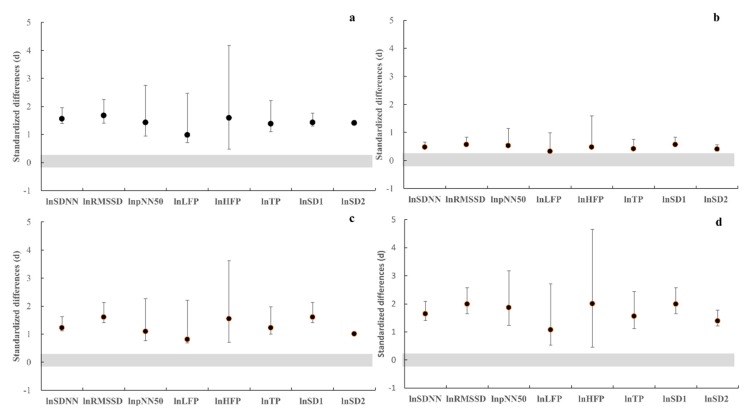
The effect size with 90% confident intervals of pre-and-post percent change in heart rate variability indices of natural logarithm values. (**a**) Yo−Yo Intermittent Recovery Test Level 1 of foot reflexology massage group; (**b**) repeated sprint ability in foot reflexology massage group; (**c**) Yo-Yo Intermittent Recovery Test Level 1 in control group; (**d**) repeated sprint ability in control group.

The FRM group showed a small ES of pre and post percent change in natural logarithm HRV indices after the RSA exercise (ES = 0.32–0.57; Figure 4). Conversely, the results demonstrated a moderate and large magnitude of pre and post percent change in natural logarithm HRV indices in the control RSA (ES = 1.07–2.00) and YY (ES = 0.81–1.61) exercise protocols and the FRM YY exercise protocol (ES = 0.99–1.59; Figure 4).

## 4. Discussion

This study is the first to report the effects of FRM on autonomic nervous function after short-term repeated bouts of sprint and incremental interval running. The main findings in the present study were that the pre-and-post exercise percent change of HRV indices in the FRM group was significantly greater during the RSA exercise protocol than during the YY exercise protocol. In addition, the FRM group showed significant improvement in %MeanRR, %SDNN, %pNN50, %RMSSD, %nHFP, %TP, %SD1 and %SD2 compared to the control group during the RSA exercise protocol. No benefits of FRM on post-exercise autonomic nervous modulation were observed after the YY exercise protocol. Our results demonstrated that FRM could result in the positive benefit of HRV modulation after repeated bouts of sprints, but not after aerobic-based incremental intermittent running.

The FRM group showed a significant difference in post-exercise changes in parasympathetic activation and sympatho-vagal balance after RSA performance but not after aerobic intermittent interval running with incremental speeds. In contrast, the control group showed similar post-exercise changes in HRV modulation between RSA and YY exercise protocols. The positive benefit of FRM in HRV modulation was also evidenced by the pre-and-post percent change and small magnitude of ES in all-natural logarithm of HRV indices in our study. These findings indicated that the FRM could contribute to an increase in parasympathetic activation and sympatho-vagal balance after the RSA exercise. Our finding was supported by a previous study showing a positive effect of sports massage on HRV modulation after a set of anaerobic exercises (jump and 30-s maximal effort of cycling test) [24]. The benefits of FRM on vagal modulation were similar to our previous findings investigating the post-intervention effect of FRM on autonomic function in patients with coronary artery disease [12].

As shown in Table 1, the physiological and psychological stresses of the participants were different between the exercise protocols but were similar between the groups. The discrepancy of perceived and physiological strains between exercise protocols may be a potential factor that influenced the outcomes of the present study. A review article with meta-analysis showed that the effectiveness of massage treatment was feasible after strenuous exercise, even though a few minutes of short-term massage was employed [7]. Our finding in the YY exercise protocol was not consistent with our hypotheses because the FRM effect on post-exercise autonomic modulation after the YY test was minor. Thus, the absence of a FRM effect was not related to the exercise intensity. Another possibility that might be related to the absence of an FRM effect on HRV during the YY exercise protocol was recovery duration. Preceding submaximal exercise intensities [25] and repeated bouts of sprint [26] can influence the recovery of autonomic activation in 10 min. However, this determinant could be prevented in our study because of the 30-min intervention or control period prior to post-exercise HRV measurement. The physiological mechanisms underlying this phenomenon remain unclear in the present study.

Application of post-exercise intervention depends upon the availability and accessibility of resources. The present study reported the positive benefits of autonomic nervous function after the anaerobic repeated-sprint exercise. The RSA exercise protocol involves all-out effort sprint and change of direction. Strength and conditioning coaches and practitioners working with repeated sprint training may consider the use of FRM to enhance the parasympathetic tone of the football players after training as an exercise recovery strategy. The FRM can be applied to football players as an alternative method of massage modality immediately after repeated sprints exercise. Future studies are needed to examine the outcome of FRM on autonomic nervous function after regular football training sessions and competitions.

## 5. Conclusions

Parasympathetic activity and sympatho-vagal balance after repeated sprint performance can be immediately increased by a FRM intervention in collegiate football players. The acute benefits of FRM on cardiac parasympathetic activity and sympatho-vagal balance can be considered as an exercise recovery strategy in sports.

## Figures and Tables

**Table 1:** Descriptive data of peak heart rate during exercises, blood lactate concentration, rating of perceived exertion and exercise performance of the participants.

Physical and Physiological Profiles	Foot Reflexology Massage (n = 14)	Control (n = 12)	*p*-Value
**RSA Exercise Protocol**			
RSA_total_ (s)	45.6 (44.5–46.3)	45.9 (44.9–48.2)	0.449
RSA_slowest_ (s)	7.8 (7.6–7.9)	7.9 (7.7–8.3)	0.317
RSA_fastest_ (s)	7.4 (7.1–7.5)	7.5 (7.2–7.8)	0.542
RSA_decrement_ (%)	−3.7 (−4.6–−2.6)	−3.5 (−6.6–−2.6)	0.826
Peak HR response (bpm)	170.0 (162.8–178.0)	171.0 (160.0–178.0)	0.961
Pre-exercise BL (mmol/L)	1.9 (1.5–2.5)	2.1 (1.7–2.7)	0.769
Post-exercise BL (mmol/L)	9.9 (9.2–13.9)	9.8 (9.3–11.6)	0.366
First lap RPE	7.0 (7.0–10.0)	7.0 (7.0–9.5)	0.510
Last lap RPE	15.0 (10.0–16.0)	15.0 (13.3–20.0)	0.161
**YY Exercise Protocol**			
Total covered distance (m)	1300.0 (1080.0–1640.0)	1020.0 (810.0–1260.0)	0.074
Peak HR response (bpm)	190.0 (185.0–193.0)	185.0 (176.0–1948.0)	0.340
Pre-exercise BL (mmol/L)	1.8 (1.4–2.1)	1.8 (1.5–2.3)	0.777
Post-exercise BL (mmol/L)	13.0 (10.6–16.0)	14.0 (12.0–14.9)	0.938
First lap RPE	7.0 (6.0–7.0)	7.0 (6.3–8.5)	0.434
Last lap RPE	20.0 (20.0–20.0)	20.0 (20.0–20.0)	0.412

RSA = repeated sprint ability; YY = Yo−Yo Intermittent Recovery Test Level 1; s = seconds; % = percent; m = metre; BL = blood lactate concentration; RPE = rate of perceived exertion. bpm = beats per minute; mmol/L = millimole per litre.
